# Increased Bone Resorption during Lactation in Pycnodysostosis

**DOI:** 10.3390/ijms22041810

**Published:** 2021-02-11

**Authors:** Ineke D.C. Jansen, Socrates E. Papapoulos, Nathalie Bravenboer, Teun J. de Vries, Natasha M. Appelman-Dijkstra

**Affiliations:** 1 Department of Periodontology, Academic Centre for Dentistry Amsterdam (ACTA), University of Amsterdam and Vrije Universiteit Amsterdam, Gustav Mahlerlaan 3004, 1081 LA Amsterdam, The Netherlands; ineke.jansen@acta.nl (I.D.C.J.); teun.devries@acta.nl (T.J.d.V.); 2 Center for Bone Quality Department of Internal Medicine division of Endocrinology, Leiden University Medical Center, 2333 ZA Leiden, The Netherlands; s.e.papapoulos@lumc.nl; 3 Department of Clinical Chemistry, Amsterdam University Medical Center, Vrije Universiteit, 1081 HV Amsterdam, The Netherlands; n.bravenboer@amsterdamumc.nl

**Keywords:** pycnodystostosis, bone remodeling, osteoclasts, cathepsin K, lactation, osteocytes

## Abstract

Pycnodysostosis, a rare autosomal recessive skeletal dysplasia, is caused by a deficiency of cathepsin K. Patients have impaired bone resorption in the presence of normal or increased numbers of multinucleated, but dysfunctional, osteoclasts. Cathepsin K degrades collagen type I and generates N-telopeptide (NTX) and the C-telopeptide (CTX) that can be quantified. Levels of these telopeptides are increased in lactating women and are associated with increased bone resorption. Nothing is known about the consequences of cathepsin K deficiency in lactating women. Here we present for the first time normalized blood and CTX measurements in a patient with pycnodysostosis, exclusively related to the lactation period. In vitro studies using osteoclasts derived from blood monocytes during lactation and after weaning further show consistent bone resorption before and after lactation. Increased expression of cathepsins L and S in osteoclasts derived from the lactating patient suggests that other proteinases could compensate for the lack of cathepsin K during the lactation period of pycnodysostosis patients.

## 1. Introduction

Lactation presents a challenge to the female skeleton to provide adequate calcium to breast milk. In contrast to other species, dietary calcium intake plays a minor role in calcium homeostasis of lactating women and milk calcium is almost exclusively obtained from bone [1]. This is achieved by upregulation of osteoclastogenesis and osteoclast-dependent bone resorption as well as by osteocytic osteolysis, the process by which osteocytes acquire certain osteoclast functions, for instance by remodeling their environment [1,2]. The abovementioned processes, which result in significant but reversible bone loss, are the combined effect of estrogen deficiency and increased production of parathyroid hormone-related peptide (PTHrP), which causes bone demineralization and degradation of its organic matrix [3]. The latter is accomplished primarily by cathepsin K, a cysteine protease that is abundantly expressed in osteoclasts and is essential for the degradation of collagen type I and other bone matrix proteins [4]. In addition, increased expression of cathepsin K has been described in osteocytes of lactating mice [5], and selective ablation of the enzyme in osteocytes of such mice prevented the increase in osteocyte lacunar area seen during lactation [6].

Cathepsin K, expressed either in osteoclasts or in osteocytes, degrades collagen type I and generates fragments such as the N-telopeptide (NTX) and the C-telopeptide (CTX) that can be quantified by immunoassays in serum and urine. The detection of these peptides is considered a reliable tool for assessing bone resorption in clinical practice and research [7,8]. Levels of these telopeptides are increased in lactating women and are associated with increased bone resorption; they normalize after weaning and the resumption of menses [1]. Although these findings are in general agreement with animal data, there is no information about the consequences of cathepsin K deficiency in lactating women.

We addressed this question and we report here blood and urine measurements and in vitro studies using osteoclasts derived from blood monocytes of a patient with pycnodysostosis during lactation and after weaning.

Pycnodysostosis, a rare autosomal recessive skeletal dysplasia, is due to loss-of-function mutations of the *cathepsin K* gene [9,10] and is characterized by short stature, osteosclerosis, acroosteolysis of the distal phalanges, and increased bone fragility. Patients have impaired bone resorption in the presence of normal or increased numbers of multinucleated, but dysfunctional, osteoclasts. These osteoclasts were shown to have the capacity to demineralize the bone, but bone matrix remained present next to the ruffled border of these cells [11]. Consistent with these histological findings, patients with pycnodysostosis have normal serum tartrate-resistant acid phosphatase (TRAcP)-5b values, a marker of osteoclast number, whereas their serum NTX and CTX values are low.

## 2. Results

### 2.1. A Lactating Woman with Pycnodysostosis

Clinical and radiographic findings of a 39-year-old patient with pycnodysostosis have been previously described [12]. In brief, the diagnosis was established at the age of 16 years and the patient has been followed at irregular intervals at the Center for Bone Quality of the Leiden University Medical Center. She has a homozygous missense mutation in exon 6 of the *CTSK* gene c.746T>C (p.Ile249Thr) and typical phenotypic and radiographic features of pycnodysostosis but she never sustained a fragility fracture. At the age of 25 years, serum markers of bone formation Alkaline phosphatase and N-terminal propeptide of procollagen type 1 (AP and P1NP) were normal, whereas the urinary hydroxyproline level (marker of bone resorption) was below the lower limit of the normal range (84.4 μmol/24 h). Histomorphometric evaluation of a transiliac bone biopsy revealed high bone volume (BV/TV 69.3%) and trabecular thickness (450 μm), compatible with the radiographic osteosclerosis. The presence of double tetracycline labels indicated normal bone formation. At the age of 34 years she delivered her first child, which she breastfed when she was seen in the outpatient clinic at three and five months postpartum while she was still lactating; she had no specific complaints. A serum analysis revealed high levels of serum CTX (1.020 and 0.903 ng/mL; upper limit of normal 0.573 ng/mL) and P1NP (188 and 168 ng/mL; upper limit of normal 59 ng/mL). Renal function was normal, as were all biochemical parameters of calcium metabolism (Table 1). When the patient was seen again after weaning, the values of bone turnover markers had returned to normal; serum CTX was 0.367 ng/mL and P1NP was 49 ng/mL.

This lactating patient with lifelong cathepsin K deficiency therefore showed for the first time in her life a normal increase in bone resorption during lactation that was also coupled with increased bone formation. To study potential mechanisms related to this response we assessed osteoclast formation and function from peripheral blood monocytes of the patient during and after lactation.

### 2.2. Osteoclast Formation and Activity In Vitro

**Figure 1:**
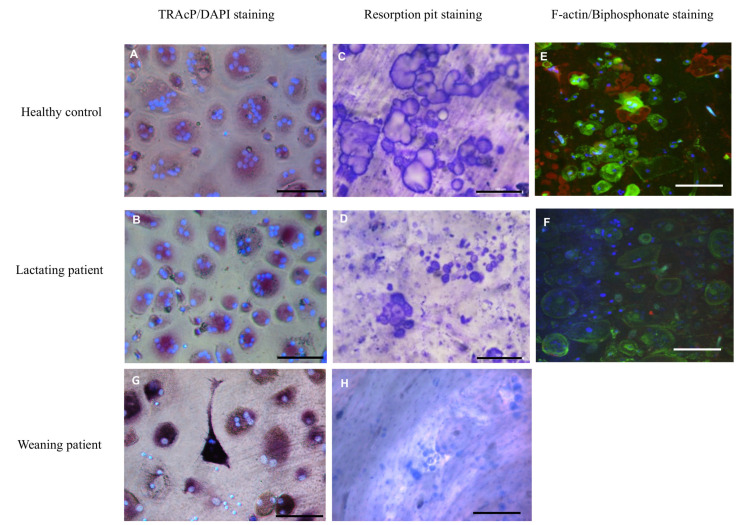
Osteoclast cultures. Osteoclasts were generated from CD14^+^ cells with MCSF and RANKL for 4 weeks and subsequently fixed and stained for TRAcP (red); nuclei were visualized with DAPI (blue). Cells from both the healthy control (**A**) and the lactating patient (**B**) generated multinucleated osteoclasts. Control osteoclasts cultured on trabecular bone slices generated resorption trenches (**C**). This was in contrast to the osteoclasts of the patient where only small round resorption pits were visible (**D**). Confocal microscopy showed that actin rings, visualized with phalloidin-alexa488 (green), were abundantly present in cells of the control (**E**). These actin rings were hardly visible in cells of the patient (**F**). Staining the resorption area with alexa-647-labeled bisphosphonate (red) revealed less resorption by cells of the patient compared to the control (Figure 1E compared to Figure 1F). The nuclei were stained with DAPI (blue). TRAcP staining and resorption of osteoclasts from the patient during weaning is displayed in **G**,**H**. Scalebar 100 µm.

CD14^+^ blood monocytes, as osteoclast precursors, were isolated from peripheral blood mononuclear cells (PBMCs) of the patient during the fifth month of lactation and after weaning. At the same two time points, blood from an age-matched postpartum healthy woman was collected. Cells were treated with M-CSF and RANKL, were cultured for four weeks either on plastic or on bovine cortical bone slices, and were stained for TRAcP activity. Multinucleated TRAcP^+^ cells with three or more nuclei were considered osteoclasts. In the cultures on plastic, similar numbers of osteoclasts were formed from CD14^+^ cells of the healthy control and the patient (Figure 1A,B,G). There were, however, remarkable differences both in the activity and structure of osteoclasts between the two. As shown in Figure 1C, osteoclasts from the healthy donor formed resorption trenches on cortical bone slices, whereas osteoclasts from the patient showed resorption pits but no trenches (Figure 1D,H). Trench formation has been associated with high collagenolytic activity, higher cathepsin K expression, and deeper demineralization [13,14].

In addition, actin rings and resorption pits, as visualized by labeled bisphosphonate, were present on the slices with osteoclasts from the control subject (Figure 1E), whereas hardly any actin staining or smaller resorption pits were visible on bone slices with the patient’s osteoclasts (Figure 1F). These differences were present during and after the lactation period. Overall, the number of osteoclasts formed on bone slices during lactation was significantly lower in the patient compared to the control.

**Figure 2:**
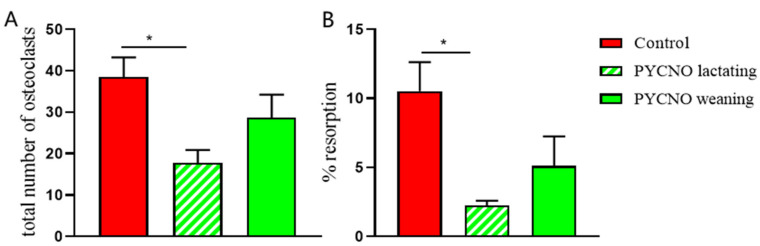
The total number of osteoclasts and resorption. (**A**) The number of osteoclasts was counted after 4 weeks of culture and the total number of osteoclasts was assessed. During the lactation period a lower number of osteoclasts was found in the PYCNO group compared to the control. * *p* < 0.05. (**B**) The from CD14^+^ cells generated osteoclasts were cultured on trabecular bone slices and resorption area was measured. The area resorbed by the PYCNO is significantly smaller compared to the control. * *p* < 0.05.

After lactation the number of osteoclasts was comparable to that of the healthy control (Figure 2A). However, in the area resorbed by the patient’s osteoclasts during and after lactation, the values were lower than those of the control subject at both time points (Figure 2B), but only significantly lower during lactation and were visible as small round pits without any trenches.

### 2.3. Expression of Cathepsin- and Osteoclast-Specific Genes

**Figure 3:**
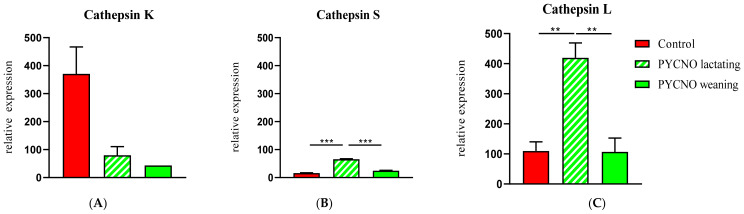
qPCR of cathepsins K, S, and L. Cathepsin K expression was very low in the patient (**A**). During lactation the expression of cathepsin L and S (**B**,**C**) was significantly elevated in the osteoclasts of the PYCNO patient. ** *p* < 0.01; *** *p* < 0.0001.

In order to provide a possible mechanism for the observed resorption, we assessed whether other proteinases had increased expression in the pycnodysostosis patient samples. Because of similarities of cathepsin K to other cysteine proteinases such as cathepsins L and S, we examined expression of the genes encoding for cathepsin L and cathepsin S. These have collagenolytic activity and their structure closely resembles that of cathepsin K [15]. *Cathepsin K* mRNA expression was low in the patient both during lactation and after weaning (Figure 3A). The expression of *cathepsin L* and *S* mRNA, however, was significantly enhanced during lactation. After weaning the levels decreased and proved to be similar to those of the control subject (Figure 3B,C). The magnitude of expression as well as of the change was much greater for *cathepsin L* than for *cathepsin S*.

The expression of *TRAcP* and *RANK* was comparable in the patient during and after lactation and not different from the expression in the control subject (results not shown).

## 3. Discussion

The present case study revealed a physiological, unexpected increase in bone resorption in a lactating woman with genetically determined cathepsin K deficiency. This increase was observed at three and five months of full-time lactation and the values of the marker of bone resorption, serum CTX, were similar to the mean value previously reported in healthy lactating women 12 to 14 weeks after delivery [8]. Levels of biochemical markers of bone resorption are typically low in patients with pycnodysostosis [16] and do not change in response to teriparatide (PTH 1-34) [17] but we did not identify any reports of lactating women with pycnodysostosis. Although the rarity of pycnodysostosis, with an estimated prevalence of about 1 to 1.7 per million [18]), may be responsible for the lack of such information, it is notable that bone markers were not even mentioned in extended reviews of the disease up until some of the most recent ones [18,19,20] nor in two brief reports of successful pregnancies [21,22]. The observed transient nature of the increase in bone resorption with normalization of serum CTX values after weaning in our patient confirm also with observations in healthy women [1]. Our report is therefore unique in probably being the first one reporting bone turnover markers during lactation in pycnodysostosis.

The second important observation in our patient was the concomitant increase in the marker of bone formation serum P1NP, indicating the coupling of bone resorption with bone formation during lactation. In a prospective cohort study of healthy lactating women, Cainero et al. [23] found significant increases in both serum CTX and P1NP compared with non-lactating controls. This suggests that unbalanced bone remodeling is not the cause of the rapid and profound, particularly trabecular bone loss in lactating women [1]. The coupling of bone resorption with bone formation in a patient with pycnodysostosis was an unexpected finding and might be difficult to explain.

Deletion of the *cathepsin K* gene (*Ctsk*) in mice decreased bone resorption but it increased bone formation. In a series of elegant experiments [6], Lotinum et al. showed that in the absence of cathepsin K in osteoclasts, bone resorption and formation are not coupled. It was shown that there are signals from osteoclasts to the osteoblast lineage (e.g., sphingosine-1-phosphate) that maintain bone formation in the presence of decreased bone resorption. In addition, matrix-derived factors that can stimulate bone formation (e.g., insulin growth factor 1, IGF-1) and are not degraded due to the lack of cathepsin K may contribute to the uncoupling of bone resorption and bone formation [24]. In support of these observations, pharmacological inhibition of cathepsin K in animals and humans decreased bone resorption, whereas bone formation was not affected [25,26,27]. It appears thus that in our patient lactation not only stimulated the pathologically reduced bone resorption characteristic of pycnodysostosis but also instituted coupling bone resorption with bone formation. The mechanism responsible for this is unknown but may be related to IGF-1 release by the degraded bone matrix.

In an attempt to better understand the findings in our patient, we performed a series of in vitro studies of the formation and activity of osteoclasts during and after lactation. The results showed that osteoclasts were formed at both time points and that these cells caused some bone resorbing activity. The number of osteoclasts was not significantly different from the healthy control, lactation or weaning. The shape of the resorption pits formed on cortical bone, however, they were small and shallow when formed from the patient’s derived osteoclasts. In addition, there was no evidence of resorption trenches in the culture of osteoclasts derived from the control subject. Pits (rounded resorption cavities) and trenches (longitudinally extended resorption lacunae) have long been known to exist in vivo and it has been postulated that the type of resorption cavities formed by osteoclasts in vitro is relevant to in vivo resorption patterns and that cathepsin K-driven collagen degradation is an absolute prerequisite for the formation of trenches [28]. Though not measured, it seems feasible that due the huge size differences between the trenches formed by the control and the tiny pits formed in the case of pycnodysostosis, the latter were much shallower. In a recent study the same group investigated differences between pit- and trench-resorption modes by osteoclasts formed in cultures of human CD14^+^ monocytes, similar to the method used in our study [13]. They found a highly significant correlation between the levels of active cathepsin K and the prevalence of trench formation in a cohort of 14 donors and confirmed the importance of cathepsin K in the switch from pit to trench mode. Their conclusions about the effects of cathepsin K in the formation of different types of resorption cavities—based on experiments in which cathepsin K levels in normal osteoclasts were modified by treatment with the cathepsin K inhibitor odanacatib—are in agreement with the direct demonstration of the inability of cathepsin K-deficient human osteoclasts to form trenches on bovine bone slices reported here. However, CTX concentrations have not been measured in the culture media, not in the study of Borggaard et al. [13] nor in our study. Such a measurement was, however, reported in a study of a 55-year-old woman with pycnodysostosis and psoriatic arthritis [29]. Multinucleated osteoclast-like cells differentiated from the patient’s peripheral blood monocytes were cultured on dentine slices to assess their ability to form resorption pits. The differences in resorption pits formed between the osteoclasts of the cathepsin K-deficient patient and those of the control subject were very similar to our observations. Importantly for our discussion, the patient had very low serum CTX levels and in the dentine disc cell-culture media only minimal levels of CTX were released. Thus, the concentration of CTX in the blood of this patient with pycnodysostosis reflected the concentration released from bone during osteoclastic bone resorption.

There might be an additional role for osteocyte-mediated degradation of bone matrix in the absence of cathepsin K. Based on the in vitro results, it is unlikely that cathepsin K-deficient osteoclasts were the only source of the increased levels of CTX in our lactating patient with pycnodysostosis. The process of bone resorption includes the degradation of insoluble collagen type I fibers, which constitute 90% of organic bone matrix. Except for cysteine proteases, matrix metalloproteinases (MMPs) have also been reported to participate in bone resorption either alone or in concert with other collagenolytic enzymes [4,30,31,32,33]. However, MMPs degrade collagen at distinct sites, operate optimally at neutral pH, and most importantly, none of the bone resorption-related MMPs (MMP-1, -2, -9, -13, and -14) is able to generate CTX from bone collagen [8,34]. Thus, independently of the relative importance of MMPs on bone resorption, these enzymes could not have been the cause of increased values of serum CTX in our patient.

Having excluded osteoclast-mediated bone resorption, cathepsin K, and MMPs as contributors to our findings, the potential role of osteocytes appears to be the most relevant. Besides being the orchestrators of bone remodeling, osteocytes are also able to modify their surrounding extracellular matrix by specialized molecular remodeling mechanisms independent of osteoblasts and osteoclasts and have the capacity to remove and replace their perilacunar and pericanalicular matrix under healthy calcium-demanding conditions such as lactation and hibernation [35,36,37,38]. In 2012 Qing et al. [5] made the fundamental observation that lactating mice had significantly larger osteocyte lacunae in the tibia, femur, and vertebra, a finding that was quickly reversed after weaning. Lactation therefore appears to stimulate a transient osteocytic perilacunar remodeling to meet increased calcium demands. These changes were associated with stimulation of the expression by osteocytes of osteoclast-specific genes, including cathepsin K and tartrate-resistant acid phosphatase. More recently, [6] Lotinum et al. showed that ablation of *Ctsk* prevented the increase in the osteocyte lacunar area, osteoclast numbers, and bone resorption without significant changes in either serum levels of P1NP or CTX between genotypes during lactation. Although these results confirmed the importance of osteocyte-controlled perilacunar bone remodeling and cathepsin K during lactation, they also underlined differences that may occur between lactating mice and lactating humans with cathepsin K deficiency [1]. Osteocyte-regulated resorption could, therefore, be the source of CTX in our patient and the study of the expression of different cathepsins in vitro provides a possible mechanistic pathway to explain bone resorption by the in vitro-generated osteoclasts from the patient.

Next to the changes of bone turnover markers in vivo, a nearly four-fold increase in the expression of cathepsin L by osteoclasts derived from the lactating patient and to a lesser extent an increased expression of cathepsin S was observed. Cathepsin L is a cysteine protease that is also expressed by osteoclasts, and before the discovery of cathepsin K it was considered the main protease responsible for bone collagen degradation during osteoclastic bone resorption [39,40]. Later evidence indicated that cathepsin L appears to participate and contribute cooperatively with other proteases to osteoclast-mediated proteolytic degradation of organic bone matrix [31] and circulating cathepsin L levels measured by ELISA were significantly correlated with the marker of bone resorption NTX in patients with osteoporosis and controls [41]. A compensatory increase in *cathepsin L* mRNA has been reported in cathepsin K-deficient mice [42], which may represent a potential rescue mechanism of a severe osteopetrotic phenotype combined with the limited activity of cathepsin K-deficient osteoclasts.

In conclusion, in this lactating patient with life-long cathepsin K deficiency we show a normal increase in bone resorption during lactation that was also coupled to increased bone formation. These novel observations in the patient with pycnodysostosis described here provide new insights into the regulation of bone turnover by cathepsins during lactation and raise questions about osteocyte-regulated bone metabolism that warrant further investigation.

## 4. Materials and Methods

Oral and written informed consent was obtained from the patient as well as the healthy control, protocol numbers END009 and W2019.028.

### 4.1. Serum Biochemistry and Bone Turnover Markers

Serum calcium adjusted for albumin binding, phosphate, and creatinine were measured by semiautomated techniques. Serum alkaline phosphatase activity (AP) was measured using a fully automated P800 modulator system (Roche BV, Woerden, Holland). PTH and 25-OH vitamin D (25-OHD) were measured using the Immulite 2500 assay (Siemens Diagnostics, Breda, Netherlands) and the LIAISON^®^ 25-OH Vitamin D TOTAL assay (DiaSorin S.A./N.V., Brussels, Belgium), respectively. Procollagen type 1 amino-terminal propeptide (P1NP) and carboxy-terminal cross-linking telopeptide of type I collagen (CTX) were determined by the E-170 system (Roche BV). PTHrP was measured in a central laboratory in the Netherlands that uses an IRMA from Diagnostics Systems Laboratories (DSL).

### 4.2. Isolation of Osteoclast Precursors from Blood

CD14^+^ blood monocytes as osteoclast precursors were isolated from human peripheral blood mononuclear cells (PBMCs) [43]. Briefly, blood from the patient and the healthy age-matched control was collected in heparin tubes, which were processed the next day. The blood was diluted with PBS containing 1% citrate (1:1) and spun down (800 g for 30 min, without breaks) in lymphoprep (Elitech, Puteaux, France) gradient solution. The resulting interphase containing peripheral blood mononuclear cells (PBMCs) were collected and washed with 1% citrate in PBS before they were passed through a cell strainer (40 µm Greiner Bio-One Monroe, NC) to ensure the recovery of a pure mononuclear cell population. The cells were counted (Muse cell counter, Merck, Darmstadt, Germany), and the cell pellet was resuspended in 80 µl buffer (PBS containing 0.5% BSA and 2 mM EDTA) for 10^7^ cells. A total of 20 µL of CD14-magnetic beads was added to the cell suspension MACS microbeads (Miltenyi Biotech, Bergisch Gladbach, Germany). According to the manufacturer’s instructions the cells and CD14-beads were mixed and incubated for 15 min at 4 °C. The column was placed in the magnetic field, rinsed, and subsequently the cell suspension was applied to the column. Unlabeled cells passed through; the column was then removed from the magnet and CD^+^ cells were flushed out and collected.

### 4.3. Osteoclast Generation

The isolated CD14^+^ cells were plated in 96-well plates (Cellstar, Greiner Bio-One) on plastic and on bovine cortical bone slices (650 µm thick) at a density of 1.10^5^ cells/well. Cells were cultured for 21 days in α-MEM (Gibco, Paisley, UK) supplemented with 5% fetal calf serum (HyClone, Logan, UT), 100 U/mL penicillin, 100 µg/mL streptomycin, and 250 ng/mL amphotericin B (Antibiotic Antimycotic solution, Sigma, St. Louis, MO) for 3 days with 25 ng/mL human recombinant M-CSF (R & D systems, Minneapolis, MN, USA). After 3 days the concentration of M-CSF was reduced to 10 ng/mL and combined with 2 ng/mL recombinant RANKL (R & D systems) until the end of the culture period. During culture the cells were maintained at 37 °C and 5% CO_2_ and the culture medium was refreshed every 3–4 days. After 28 days of culture, the wells were washed with PBS and fixed in 4% PBS buffered formaldehyde, stored at 4 °C, and used for tartrate-resistant acid phosphatase (TRAcP) staining, The bone slices were stored in MilliQ water at 4 °C for bone resorption visualization.

### 4.4. Tartrate Resistant Acid Phosphatase (TRAcP) Staining

The cells were stained for TRAcP using the acid phosphatase leukocyte (TRAcP) Kit from Sigma (Sigma-Aldrich, St. Louis, MO, USA) according to manufacturer’s instructions; only a higher KNa-tartrate solution was used (1.0 M) to block all non-tartrate-resistant acid phosphatases. The nuclei were visualized with 4′6-diamino-2-phenylindole dihydrochloride (DAPI). Multinucleated TRAcP^+^ cells with three or more nuclei were considered osteoclasts and were counted on bone in standardized fields and on plastic per well using a combination of light and fluorescence microscopy (Leica DFC320; Leica Microsystems, Wetzlar, Germany).

### 4.5. Resorption Pit Staining

Resorption was measured on slices of bovine cortical bone 650 μm thick and fit into a 96-well plate. CD14^+^ monocytes were cultured on these bone slices for 28 days with M-CSF and RANKL, as mentioned above. After this period, the cells were removed with 0.25 M NH_4_OH. The slices were washed in distilled water, incubated in a saturated alum (KAl(SO4)2•12H2O) solution, washed in distilled water, and stained with Coomassie Brilliant Blue. Resorption pits were visualized by light microscopy (Leica DFC320). Micrographs of the resorbed area were made with SI-17 x10 magnification. The total resorbed area was quantified using Image-Pro Plus (Media Cybernetics, Rockville, MD, USA) and are presented as a percentage of the total area.

### 4.6. Confocal Microscopy

To visualize osteoclasts and their resorption pits, CD14^+^ cells were cultured on bone slices as mentioned above. After 4 weeks of culturing, bone slices were fixed with 4% formalin. The bone slices were stored at 4 °C in PBS until they were analyzed by confocal laser scanning microscopy. Prior to visualization the cells were washed with 0.05% triton in PBS, and subsequently washed 3 times with PBS. The slices were incubated with either Phalloidin-488 (Cytoskeleton Inc., Denver, CO, USA) or labeled as bisphosphonate (RIS-alexa-647) [44,45].

This resulted in the visualization of actin filaments present in the ruffled border area and in the cell membrane of the osteoclasts as well as the resorbed area, respectively. Both phalloidin and bisphosphonate stainings were added together as a mix and incubated for 30 min at room temperature. The nuclei were stained with DAPI. All incubations were performed at room temperature. The bone slices were stored at 4 °C in PBS until they were analyzed by confocal laser scanning microscopy (Nikon A1R, Nikon, Amsterdam, The Netherlands).

### 4.7. Real-Time Quantitative PCR (qPCR)

RNA from cultured CD14^+^ cells was isolated using the RNeasy Mini Kit (Qiagen, Hilden, Germany) according to the manufacturer’s instructions. After measuring the RNA concentration with a multilabel plate reader (Synergy HT BioTek Instruments, Bad Friedrichshall, Germany), 100 ng RNA was reverse transcribed to cDNA qPCR. The reactions were performed with a LightCycler^®^ (Roche Diagnostics, GmbH, Mannheim, Germany) using 5 ng cDNA and 300 nM of each primer in a total volume of 20 μL containing SYBR Green PCR Master Mix (Green Supermix (Roche Laboratories, Indianapolis, IN, USA), following the manufacturer’s instructions. Samples were normalized for the expression of porphobilinogen deaminase (PBGD), the expression of which was not altered by the experimental conditions, by calculating the ΔCt (Ct gene of interest—Ct PBGD). The expression of the different genes is given as 2^−(ΔCt)^. The primers used for the detection of the various genes are indicated in Supplementary Table S1.

### 4.8. Statistical Analysis

All experiments were performed in triplicate. Data are expressed as mean ± SEM and were analyzed with one-way ANOVA with Tukey’s post-test using Graphpad Prism version 8 (Graphpad, San Diego, CA, USA). *p* values < 0.05 were considered statistically significant.

## Figures and Tables

**Table 1:** Biochemical parameters of a woman with pycnodysostosis during lactation and weaning.

Parameter	Lactation	Weaning	Normal Range
Creatinine	50	56	49–90 μmol/L
eGFR	>90	>90	>60 mL/min/1.73 m^2^
Calcium	2.33	2.25	2.15–2.55 mmol/L
Phosphate	1.45	1.26	0.90–1.50 mmol/L
Magnesium	0.9	0.99	0.70–1.10 mmol/L
25-hydroxy D	80	65	50–250 nmol/L
PTH	2	4.5	0.7–8.0 pmo/L
PTHrP	<0.3		<0.7 pmol/L
AP	125 *	78	<98 IU/L
P1NP	178 *	49	<59 ng/mL
CTX	0.962 *	0.367	<0.573 ng/mL

* Mean of two measurements, abnormal values.

## Data Availability

Data available on request.

## Supplementary Materials

The following are available online at https://www.mdpi.com/1422-0067/22/4/1810/s1.
