# Supplementary material for: Increased Bone Resorption during Lactation in Pycnodysostosis
Source: Int J Mol Sci. 2021 Feb 11;22(4):1810. doi: 10.3390/ijms22041810 (PMC7918824; doi:10.3390/ijms22041810)
Supplement: Supplementary file 1 [file ijms-22-01810-s001.pdf]

Supplementary Table S1:

Primer sequences used for qPCR

Primer	Sequence 5'-3'	Accessionnumber
PBGD	TgCAgTTTgAAATCATTgCTATgTC	ENSG00000113721
	AACAggCTTTTCTCTCCAATCTTAgA	
Cathepsin K	CCATATgTgggACAggAAgAgAgTT	ENSG00000143387
	TgCATCAATggCCACAgAgA	
Cathepsin L	CgAACTCTgCTggCCTTgA	ENSG00000135047
	AAAggCAgCAAggATgAgTgTAg	
Cathepsin S	CTgggAgACATgACCAgTgAAg	ENSG00000163131
	gCAATATCCgATTAgggTTTgACT	
TRAcP	CACAATCTgCAgTACCTgCAAgAT	ENSG00000102575
	CCCATAgTggAAgCgCAgATA	
RANK	CCTggACCAACTgTACCTTCCT	ENSG00000141655
	ACCgCATCggATTTCTCTgT	
